# Supplementary material for: Biological fermentation pilot-scale systems and evaluation for commercial viability towards sustainable biohydrogen production
Source: Nat Commun. 2024 May 28;15:4539. doi: 10.1038/s41467-024-48790-4 (PMC11133433; doi:10.1038/s41467-024-48790-4)
Supplement: Supplementary file 1 — Supplementary Information [file 41467_2024_48790_MOESM1_ESM.pdf]

Supplementary Information

Biological Fermentation Pilot-Scale Systems and Evaluation for Commercial Viability Towards Sustainable Biohydrogen Production

Quanguo Zhang[1,2], Youzhou Jiao[1], Chao He [1], Roger Ruan [3], Jianjun Hu [1], Jingzheng Ren[4], Sara Toiniolo[5], Danping Jiang [1,2], Chaoyang Lu [1], Yameng Li[1,2]*, Yi Man [4], Huan Zhang [1,6]*, Zhiping Zhang [1,6]*, Chenxi Xia [2], Yi Wang [6], Yanyan Jing [1,6], Xueting Zhang[2], Ruojue Lin[4], Gang Li[6], Jianzhi Yue [1] and Nadeem Tahir[6]

[1] Key Laboratory of New Materials and Facilities for Rural Renewable Energy of Ministry of Agriculture and Rural Affairs of China, College of Mechanical & Electrical engineering, Henan Agricultural University, Zhengzhou 450002, China.

[2] Institute of Agricultural Engineering, Huanghe S & T University, Zhengzhou 450006, China.

[3] Biorefining Center, University of Minnesota, Minneapolis and St. Paul, 55455, USA

[4] Department of Industrial and Systems Engineering, The Hong Kong Polytechnic University, Hong Kong.

[5] CESQA, Department of Industrial Engineering, University of Padua, Via Marzolo 9, 35131 Padova, Italy.

[6] Henan International Joint Laboratory of Biomass Energy and Nanomaterials, Collaborative Innovation Center of Biomass Energy, Henan Agricultural University, Zhengzhou 450002, China.

* Corresponding author (, Yameng Li, Huan Zhang and Zhiping Zhang) at: Key Laboratory of New Materials and Facilities for Rural Renewable Energy of Ministry of Agriculture and Rural Affairs of China, College of Mechanical & Electrical engineering, Henan Agricultural University, Zhengzhou 450002, China.

Corresponding Email: liyameng2017@163.com; zhanghuan5754@163.com;

zhangzhiping715@163.com;

Contents

Supplementary Fig.1-Research roadmap 4

Supplementary Fig.2 Biochemical process of hydrogen production 5

Supplementary Fig.3 Illustration of experiment equipment 6

Supplementary Note 1. Photosynthetic bacteria growth and hydrogen production under different light sources 7

Supplementary Fig.4. Heat energy transmission of the system 8

Supplementary Fig.5 Illustration of experiment equipment of the hydrogen production system on thermal effect 9

Supplementary Note 2. Liquid rheological properties of baffle plate reactor and multiphase flow theory 10

Supplementary Fig.6 Grid of reacting region 14

Supplementary Fig.7. The 11m^3 pilot-scale baffled continuous flow dark-photo fermentation hydrogen production reactor. 15

Supplementary Fig.8. 3D view of the reactors 16

Supplementary Note 3. The process of handling raw materials 17

Supplementary Note 4. Continuous hydrogen production 18

Supplementary Fig.9 Dark fermentation unit (a) pH (b)Oxidation reduction potential 19

Supplementary Fig.10 Photo fermentation unit (a) pH (b) Oxidation reduction potential 20

Supplementary Note 5. Life cycle assessment method 21

Supplementary Fig.11 System boundary 23

Supplementary Table.1 Life cycle inventory (1 t hydrogen) 24

Supplementary Table.2 GW from different hydrogen production technology 25

Supplementary Fig.12 Sensitivity analysis 26

Supplementary Note 6. Life cycle costing assessment method 27

Supplementary Fig.13. Transformation process of straw biomass based hydrogen production system 29

Supplementary Fig.14. Relation between NPV and the related factors 30

Supplementary Table.3. Cost of hydrogen from different hydrogen production technology 31

Supplementary Table.4. Profit and loss statement and dynamic economic analysis 32

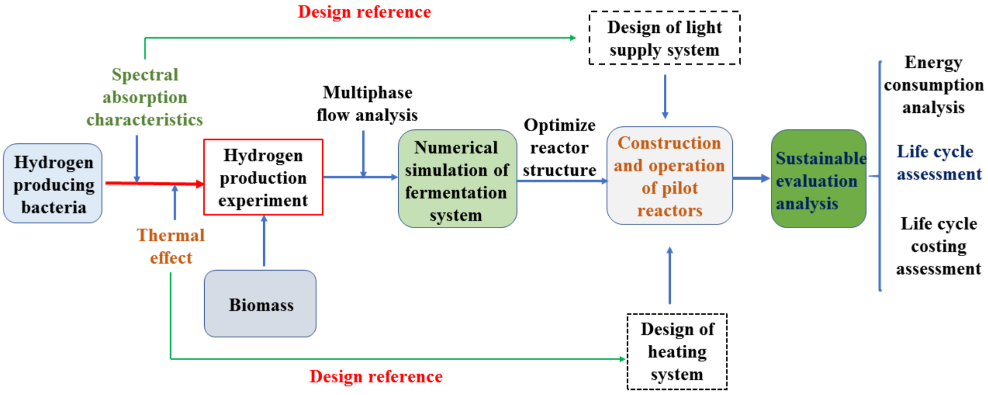

Supplementary Fig.1 Research roadmap

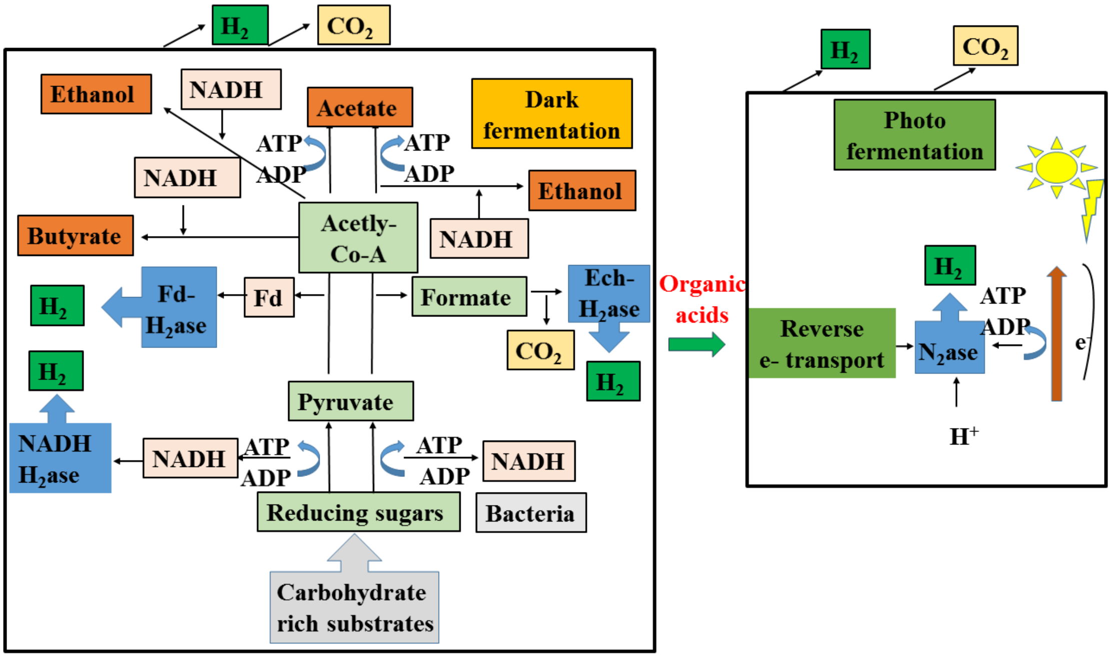

Supplementary Fig.2 Biochemical process of hydrogen production

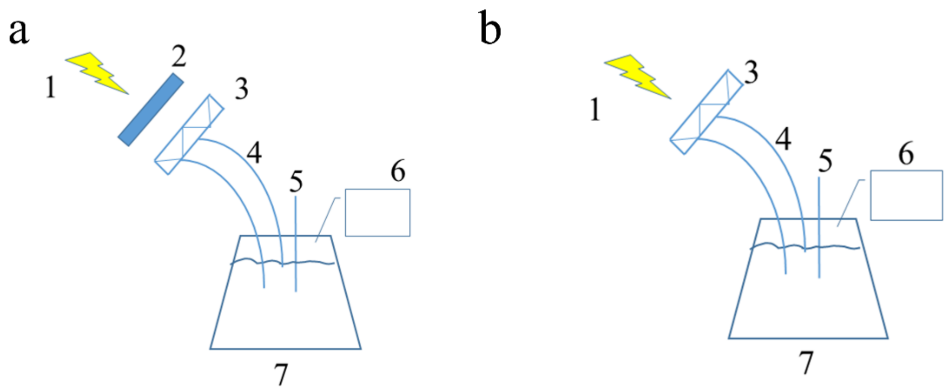

Supplementary Fig.3 Illustration of experiment equipment under different conditions. **a** With light filter. **b** Without light filter

1 Light source 2 Light filter 3 Solar collector 4 optical fiber 5 Sampling port 6 Air collecting bag 7 Reaction bottle

Supplementary Note 1. Photosynthetic bacteria growth and hydrogen production under different light sources

The light source composed of multiple LEDs with different wavelengths. As follows: yellow light source (580-596 nm), blue light source (440-475 nm), green light source (490-580 nm), red light source (615-630 nm)

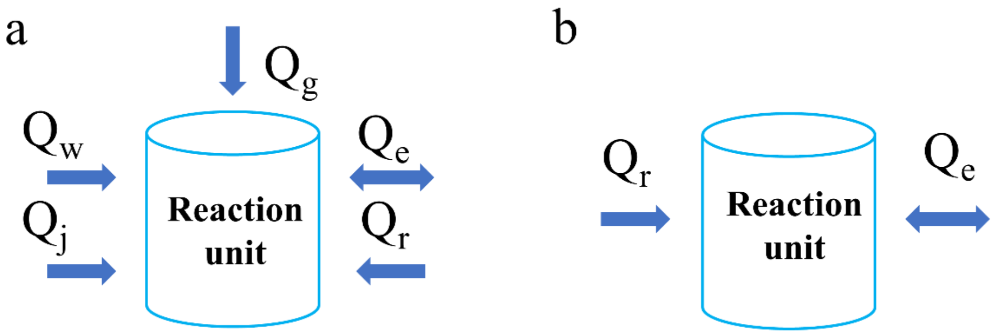

Supplementary Fig.4. Heat energy transmission of the system. **a** Photo fermentation system. **b** Dark fermentation system. (Q_g - photothermal, Q_r - heat from hydrogen production, Q_w - heat from reflection or refraction of photosynthetic bacteria, Q_j – heat from reflection or refraction of matrix, Q_e - heat transfer between the reaction system and the environment.)

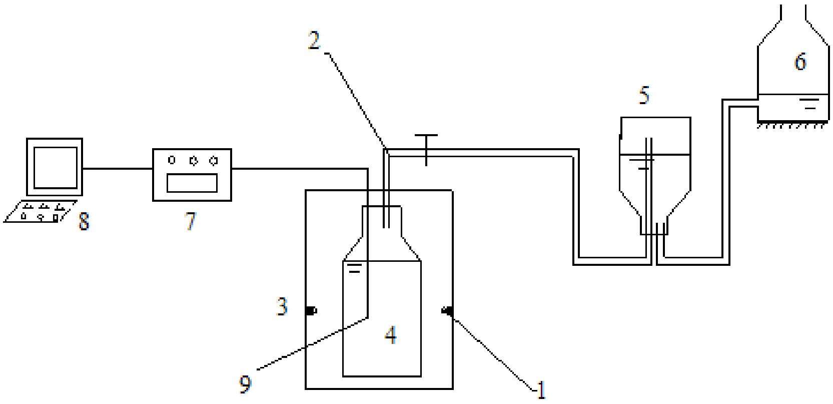

Supplementary Fig.5 Illustration of experiment equipment of the hydrogen production system on thermal effect

1 Light source. 2 Air duct. 3 Thermostat. 4 Vacuum reaction bottle. 5 Collection bottle. 6 Water storage bottles. 7 Precision digital thermometers. 8 Computers. 9 Thermometer sensors.

Note: for the dark fermentation system, 1 light source is turned off.

Supplementary Note 2. Liquid rheological properties of baffle plate reactor and multiphase flow theory

Governing equation: Due to the small density difference between the solid-liquid phases in the experiment, the particle and liquid phases have good following ability, and the mixture model can meet the calculation requirements. In this model, the interphase slip velocity is used to couple the interphase forces, and the control equation is obtained by using the mixture physical properties parameters and weighted average of the two-phase physical properties parameters. The continuity equation, momentum equation, and second phase continuity equation are as follows:

$$\nabla \cdot (\rho_m \vec{v}_m) = m \qquad \text{(S1)}$$

$$\nabla \cdot (\rho_m \vec{v}\vec{v}) = -\nabla p + \nabla\left[\mu_m\left(\nabla v_m + \nabla v_m^{-T}\right)\right] + \rho_m \vec{g} + \nabla\left(\alpha\rho_s \vec{v}_{dr,s}\vec{v}_{dr,s} + (1-\alpha)\rho_l \vec{v}_{dr,l}\vec{v}_{dr,l}\right) \quad \text{(S2)}$$

$$\nabla(\alpha\rho_s \vec{v}_m) = -\nabla(\alpha\rho_s \vec{v}_{dr,s}) \qquad \text{(S3)}$$

The slip velocity between phases is defined as follows:

$$\vec{v}_{dr,s} = \vec{v}_s - \vec{v}_m = \frac{(1-\alpha)\rho_l}{\rho_m}\vec{v}_{ls} \qquad \text{(S4)}$$

$$\vec{v}_{dr,\ l} = \vec{v}_l - \vec{v}_m \qquad \text{(S5)}$$

V_{dr}– Drift velocity, m/s; V_{ls}- relative velocity, i.e. slip velocity; the subscripts s, l, and m represent the solid phase, liquid phase, and mixed phase, respectively.

Viscosity calculation:

The particle viscosity is calculated as follows:

$$\mu_s = a\mu_{s,\ col} + b\mu_{s,\ kin} + c\mu_{s,\ b} \qquad \text{(S6)}$$

Collision part of shear viscosity:

$$\mu_{s,\ col} = \frac{4}{5\pi}\alpha\rho_s d_s g_{0,\ ss}(1+e_{ss})\sqrt{\pi\Theta_s} \qquad \text{(S7)}$$

Dynamic viscosity equation:

$$\mu_{s,kin} = \frac{10\rho_s d_s \sqrt{\pi\Theta_s}}{96\alpha(1+e_{ss})g_{0,ss}}\left[1+\frac{4}{5}g_{0,ss}\alpha(1+e_{ss})\right]^2 \quad \text{(S8)}$$

Volume viscosity formula of particles:

$$\mu_{s,\ b} = \frac{4}{3}\alpha\rho_s d_s g_{0,ss}(1+e_{ss})\left(\frac{\Theta_s}{\pi}\right)^{0.5} \quad \text{(S9)}$$

e_{ss} -Reduction coefficient of particle collision (0.9); $g_{o,ss}$- Radial distribution function; Θ_s - Particle temperature of solid phase,K.

$$g_o = \left[1-\left(\frac{\alpha}{\alpha_{s,\max}}\right)^{\frac{1}{3}}\right]^{-1} \quad \text{(S10)}$$

$$\Theta_s = \frac{1}{3}\vec{v}_s\vec{v}_s \quad \text{(S11)}$$

Ultimate stacking concentration $\alpha_{s,max}$=0.63

Boundary and initial conditions:

At the beginning of the calculation, there is a clear solid-liquid boundary inside the container, and the initial velocity of the substance in the container is 0. Driven by the inlet solid-liquid two-phase flow, turbulence disturbance begins to occur

Import conditions: F_s=0.04, F_l=0.96;

Initial condition: t=0, F_s=1, F_l=0, $0 < y < 3.5$;

F_s=0, F_l=1, $3.5 < y < 55$

The established model is a two-dimensional model. When establishing the model, the boundary at the top of the reactor is based on the reaction liquid level. GAMBIT software is used to generate a grid in the calculation. The grid in the reaction zone is shown in **Supplementary Fig.6**, with a grid spacing of 1cm and a total of 6650 nodes.

Fluent software package was used for calculation and solution, with implicit algorithm, two-dimensional spatial format, and unsteady flow; the convective phase adopts a first-order upwind scheme; the time step is 0.2s, and it is saved every 10 time steps calculated; the simple method is used for calculation.

Related assumptions:

In view of the structure and operational characteristics of the biological hydrogen production system, for the convenience of calculation and research, the following assumptions are made in this article:

(1) Simplify the bioreactor into a two-dimensional model for processing;

(2) Assuming that the entire system is in a stable environment without considering the heat production of biological reactions, i.e. the reaction temperature remains constant;

(3) Assuming that the density of the reaction liquid and solid is only related to temperature during the reaction process;

(4) The pressure change inside the reactor is relatively small, and the reaction liquid can be regarded as an incompressible fluid;

(5) Neglecting the heat dissipation of the reactor wall and the radiation heat transfer with the external environment, the reactor wall is considered as an adiabatic non sliding solid wall;

(6) The viscosity variation of each compartment in the reactor is relatively small. Assuming that the viscosity of each point in the reactor is consistent, it does not change with time during the feeding stage;

(7) The concentration distribution of feed is uniform;

(8) Only two-phase reactions are considered, and the influence of gases is no longer considered.

Calculation conditions:

(1) Size

The length of the inlet and outlet is 4 cm, the diameter is 2cm, the height of the lower part of the baffle is 6 cm, and the height of the upper part is 2 cm.

(2) Other conditions

The inlet speed is 0.3 m/s; Liquid phase density ρ= 950 kg/m^3; dynamic viscosity μ= 9.4 × 10^{-4} Pa·s; solid particle density 1275 kg/m^3; the particle size is 235 nm.

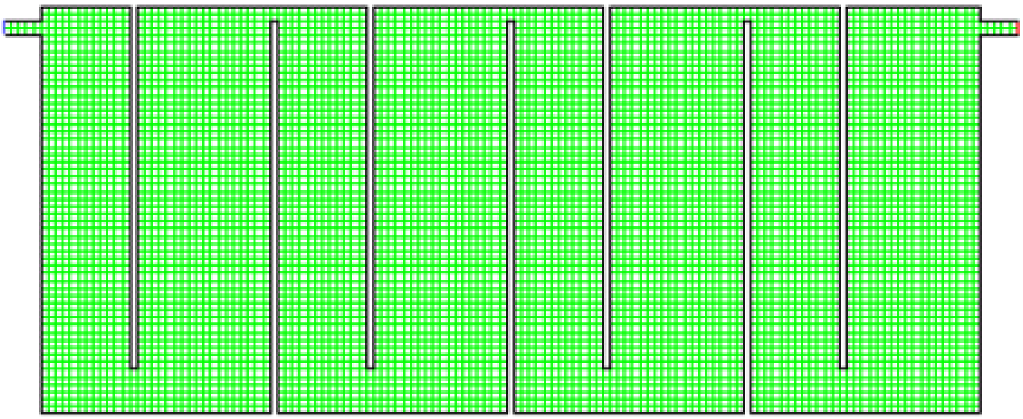

Supplementary Fig.6 Grid of reacting region

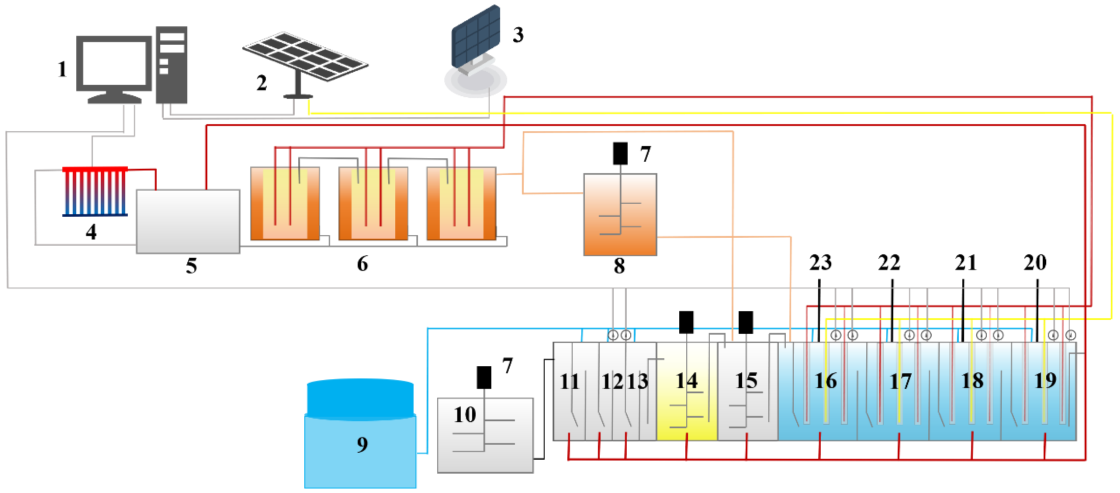

Supplementary Fig.7. The 11m^3 pilot-scale baffled continuous flow dark-photo fermentation hydrogen production reactor. 1 Intelligent control center. 2 Solar panels. 3 Solar fiber import plant. 4 Solar water heater. 5 Hot water storage tank. 6 Photosynthetic bacteria tank . 7 Agitator . 8 Photo fermentation medium tank . 9 Gas tank. 10 Dark fermentation medium tank. 11-13. The dark chambers from No. 1 to 3. 14 DFE treatment chamber. 15 Mixing chamber. 16-23 The light chambers from No. 1 to 8.

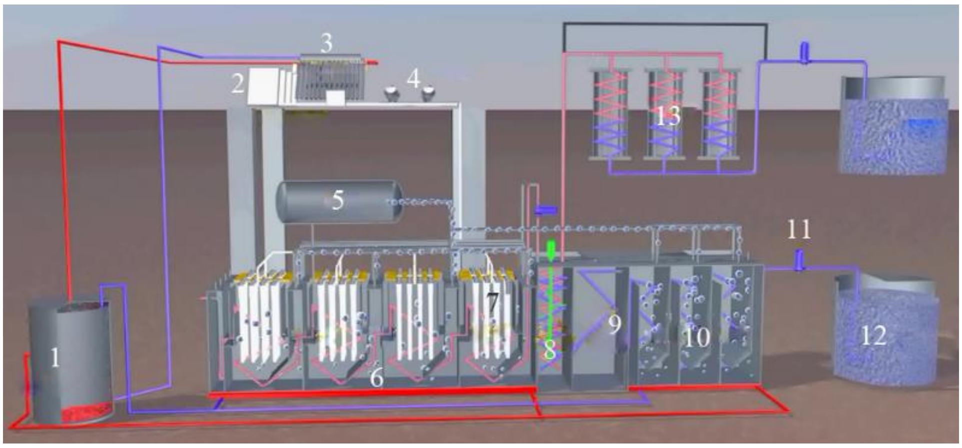

Supplementary Fig.8. 3D view of the reactors

1 Hot water storage tank. 2 Solar panels. 3 Solar water heater. 4 Solar fiber import plant. 5 Gas tank. 6 Photo fermentation unit. 7 Light pipe. 8 Mixing chamber. 9 Dark fermentation broth treatment chamber. 10 Dark fermentation unit. 11 Blender.

Supplementary Note 3. The process of handling raw materials

The particle size of cornstalk is an important physiochemical property, which can directly affect the economic performance and efficiency of converting substrate into reducing sugar. The cornstalks are first crashed into particles of 0.45-1 nm at 15 kWh/t electricity consumption (Shandong Heze KAICHUANG Machinery Company, 93ZRG-680) and then smashed with WFJ-20 microgrinder to be particles of 330-420 nm at 25 kWh/t power consumption (Jiangyin LINGLING Machinery Manufacturing Company). The pulverized cornstalks are hydrolyzed with cellulase with the ratio of solid to liquid being 1:10. Enzymolysis is carried out in pH 4.8 sodium citrate buffer solution with industrial cellulase (enzymatic load: 25 mg/g; enzymatic hydrolysis temperature: 50℃) for 48 h.[1,2] Several enzymatic hydrolysis reactors are used to make sure continuous working of fermentation plant. Around the reactors of enzymatic hydrolysis, insulation layer is used for maintaining the heat. Temperature is maintained by circulating hot water, which is heated by solar heater. The energy of heated water could satisfy the heat demand of enzymatic hydrolysis and no electric heating is needed. During enzymatic hydrolysis, 1.1 kW blender is used for intermittent stirring. The post-hydrolysis residue is left in the sunshine for drying and then used as the substrate for mushroom plantation. The solar energy for drying hydrolysis residue is not considered as consumed energy during the life cycle.

Supplementary Note 4. Continuous hydrogen production

For dark and photo-fermentation hydrogen production system, 25 g/L reducing sugar hydrolyzed from corn straw was used as substrate. During dark fermentation unit with three chambers (each of chamber has working volume of 1 m^3), the dark fermentation system was conducted under initial pH of 5.5, HRT of 12 h and 40℃. The inoculation amount of dark fermentation hydrogen producing bacteria was set to 20%(v/v)(cell dry weight 1.13±0.15 g/L). The dark fermentation effluents flowing out of the dark fermentation unit first entered the treatment tank to remove excess NH_4^+ by zeolites adsorption. And then, the treated dark fermentation effluents (< 3 mM NH_4^+) were pumped into the mixing chamber, the micronutrient solution and the C, N, P of were added in proportion, the pH was adjusted to 7.0. The mixture and inoculum (20% (v/v), cell dry weight 1.36±0.15 g/L) were injected into the photo fermentation unit by pump. The fermentation conditions were set to temperature of 30 ± 1℃, light intensity of 3000 ± 200 lx, and HRT of 24 h, respectively. The physicochemical properties of fermentation broth in different tanks are shown in **Supplementary Fig.9 and Fig.10.**

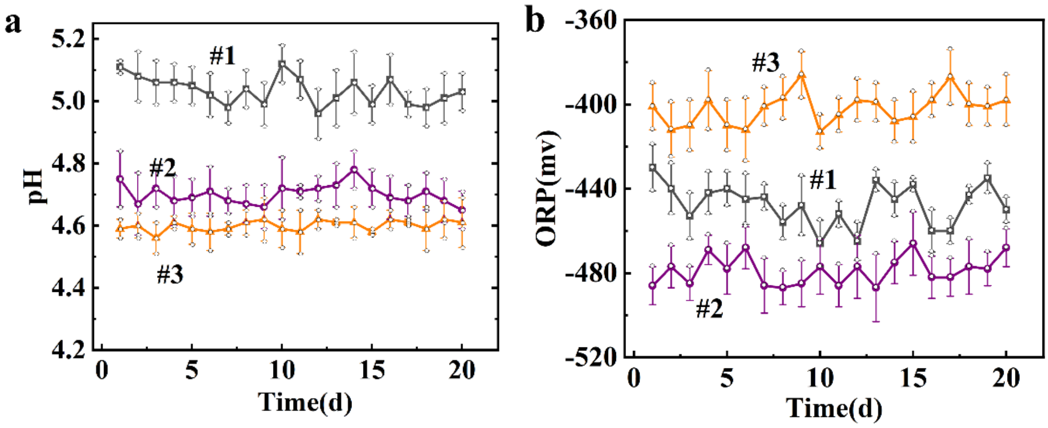

Supplementary Fig.9 Operating data of dark fermentation unit. **a** pH (n=3, independent experiments). **b** Oxidation reduction potential (n=3, independent experiments). # refers to chamber number.

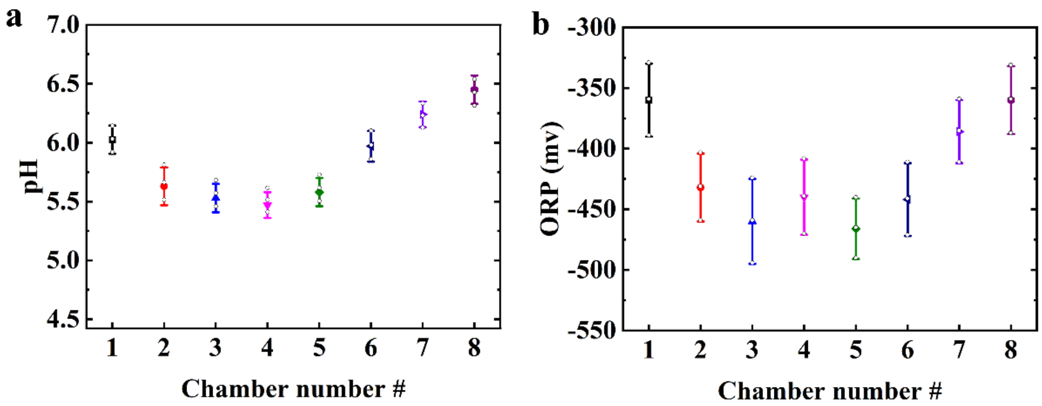

Supplementary Fig.10 Operating data of dark fermentation unit of photo fermentation unit. **a** pH(n=3, independent experiments). **b** Oxidation reduction potential(n=3, independent experiments).

Supplementary Note 5. Life cycle assessment method

The system boundary can be divided into three stages: 1. raw material pulverization; 2. enzymolysis pretreatment; 3. fermentation based hydrogen production. According to ISO 14040 and ISO 14044, LCA for hydrogen production systems is made by using SimaPro 8.5. The life cycle data are collected mostly from China and supplemented with the database in Ecoivent 3.1 so that the assessment results can be more representative in China. ReCiPe 2016 Midpiont (H), adopted in the life cycle assessment, incorporates 18 categories[3]: (1)Global warming(GW), (2)Stratospheric ozone depletion(SOD), (3)Ionizing radiation(IR), (4)Ozone formation, Human health(OFHH), (5) Fine particulate matter formation(FPMF), (6)Ozone formation, Terrestrial ecosystems(OFTE), (7)Terrestrial acidification(TA), (8)Freshwater eutrophication(FEP), (9)Marine eutrophication(MEP), (10)Terrestrial ecotoxicity(TE), (11)Freshwater ecotoxicity(FE), (12)Marine ecotoxicity(ME), (13)Human carcinogenic toxicity(HCT), (14) Human non-carcinogenic toxicity(HNCT), (15)Land use (LU), (16)Mineral resource scarcity(MRS), (17)Fossil resource scarcity(FRS), (18)Water consumption(WC).

System boundary determination:

This paper assesses hydrogen production systems, namely dark and photo-fermentation system. The boundaries of hydrogen production system are shown in **Supplementary Fig.11**, including straw pulverization, enzymolysis

pretreatment, and hydrogen production fermentation. The functional unit is 1 t H_2. Life cycle inventory is shown in **Supplementary Table.1**.

Sensitivity analysis: Supplementary Fig.12 shows the sensitivity analysis results.

As for the environmental impact in the life cycle of dark-photo fermentation, S1 has more significant decrease in IR and ME than the decrease in S2. This makes it clear that cellulase contributes more to IR and ME and the effect decreases by 6.43% and 3.17%, respectively. Its effect on other items is weakened by 0.2-2.5%. The declination in solid-to-liquid ratio renders the weakening of life cycle environmental impact on FPMF to be most significant (9.28%), followed by FEP by 8.97%, IR, OFHH, FRS and WC by 7%, OFTA, TE, FE and HNCT by 6%, and other items by 2-5%.

By comparison, it can be found that the decrease in solid-to-liquid ratio (S2) can better weaken the environmental impact in the life cycle of dark and photo-fermentation hydrogen production system.

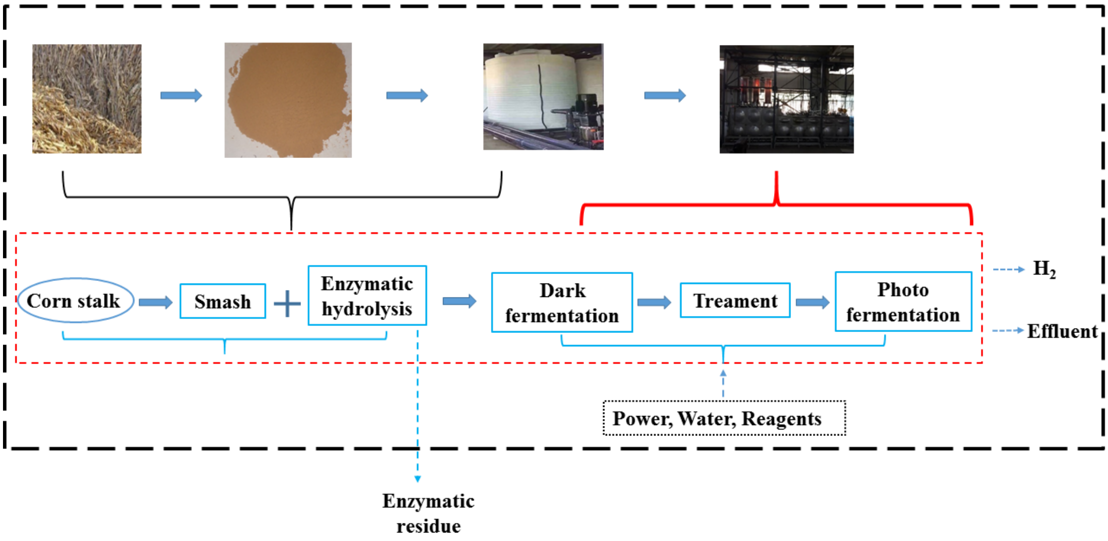

Supplementary Fig.11 System boundary

Supplementary Table.1 Life cycle inventory (1 t hydrogen)

Process	Material & Energy	Metric	Dark and photo fermentation
Raw material			
	Straw	t	40.00
Straw pulverization			
	Land occupation	m^2a	80
	Steel	kg	2.00
Enzymolysis			
	Water	t	590
	Cellulase	t	1.00
	Citric acid	t	1.93
	Sodium citrate	t	3.18
	Land occupation	m^2a	50
Fermentation			
	Water	t	290
	Land occupation	m^2a	210
	Yeast extract	t	0.65
	NaCl	t	2.72
	Peptone	t	2.56
	Dipotassium phosphate	t	0.97
	$MgCl_2$	t	0.08
	Sodium acetate	t	0.32
	Steel	t	0.51
	Polyurethane	kg	1.11

Supplementary Table.2 GW from different hydrogen production technology

Hydrogen production method	Global warming t CO_2 eq/t H_2	References
CLC-SR	10.76	[4]
Underground coal gasification	18	[5]
Deep IGCtH	30.47	[6]
Lurgi SGCtH	36.41	[6]
Biomass gasification	10.56	[7]
Coal Gasification	11.30	[8]
ESR-bioeth	9.2	[9]
SMR-F.M	11.2	[9]
SMR	15	[10]
DF-MEC	17	[11]
DPFHP	9.37	This study

CLC-SR：Chemical looping combustion thermally coupled steam reforming

Lurgi SGCtH: Lurgi surface gasification based coal-to-hydrogen

Deep IGCtH: deep in-situ gasification based coal-to-hydrogen

ESR-bioeth ：Bioethanol into hydrogen steam reforming

SMR-F.M（Fossil-methane steam reforming）

DPFHP: Dark and photo-fermentation hydrogen production

DF-MEC :Dark fermentation-microbial electrolysis cell

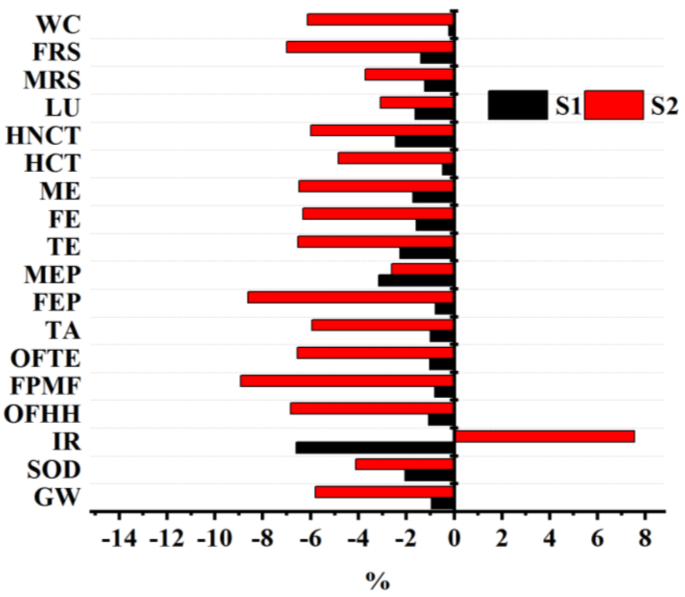

Supplementary Fig.12 Sensitivity analysis (the numerical variation is based on the original analysis results), S1: Reduction of 15% enzyme dosage, S2: Solid liquid ratio 1:8

Supplementary Note 6. Life cycle costing assessment method

Life cycle costing assessment covers all the costs arising from the whole hydrogen production stages, such as design, construction, production, operation, maintenance, and materials disposal. LCC assessment relies on the established system boundary, for change in the latter will affect the former. In this study, the LCC assessment adopts the same system boundary in LCA. The capital input for transforming biological straws into hydrogen production system in this study is listed in **Supplementary Fig.13**.

Based on a market survey, the financial and economic effect of the project are estimated according to the project construction conditions, market price at project site in 2017, average input-output level in local place, domestic statistical information, and expected market conditions.[12] The cost for producing 1 t hydrogen can be expressed in equation (S12):

$$\text{Cost} = \frac{\text{Fixed asset } (1-0.05)}{20\cdot 360\cdot \text{Hydrogen yield}} + \frac{\text{Labor cost}}{20\cdot 360\cdot \text{Hydrogen yield}} + \frac{\text{Maintenance cost}}{360\cdot \text{Hydrogen yield}} + \text{Pretreatment cost} + \text{Fermentation cost} + \text{Welfare} \qquad \text{(S12)}$$

Techno-economic assessment indicators can be categorized into static indicators and dynamic indicators. The former gives no consideration of time factor of capital and has advantage in easy calculation and understanding, however, they cannot accurately reflect the actual conditions of the investment project. The latter takes into consideration of time factor of capital, such as payback period, net present value and internal rate of return. The income and expenses of the investment project during the whole life cycle are also considered. Thus dynamic indicators are more scientific and comprehensive than the static indicators.[13]

Net present value (NPV) is the sum of present value of net cash flows over all the years discounted to the beginning of the period as per industrial discount rate

during the project computation period, as shown in equation (S13).

$$\mathrm{NPV} = \sum_{t=0}^{n}(CI - CO)_t \cdot (1 + i_0)^{-t} \qquad \text{(S13)}$$

Investment payback period (TP) is the period in which investment is paid back with capital return, as shown in equation (S14).

$$\sum_{t=0}^{T_p}(CI_t - CI_o) \cdot (1 + i_0)^{-t} \qquad \text{(S14)}$$

Internal return rate (IRR) is the discount rate when net present value is zero, as shown in equation (S15).

$$NPV = \sum_{t=0}^{n}(CI - CO)_t \cdot (1 + IRR)^{-t} = 0 \qquad \text{(S15)}$$

Where, *CI* is the cash flow, CNY; *CO* is the cash outflow, CNY; $(CI\text{-}CO)_t$ is the net cash flow in the t-th year, CNY; i_0 is the discount rate.

Profit and loss statement and dynamic economic analysis is shown in **Supplementary Table.4**.

Economic sensitivity analysis

Sensitivity analysis is a sort of uncertainty analysis approach that investigates the effect of specific change in different uncertainty factors on the economic aspect of a project. According to the production cost, for dark and photo- fermentation system, fixed asset investment, welfare and reagents consumption has the major proportion of the total production cost. The sales income of the system is significantly affected by the sales prices of hydrogen and fermentation residual liquid. According to market price at project site, the sales prices of hydrogen was set 56 CHY/kg(7.65$/kg), the fermentation residual liquid was set 20 CHY/t(2.73$/t). To observe the effect on the economic interests of the system, the range of selected parameters is set to be ±30%. The results variation of the financial NPV is as shown in **Supplementary Fig.14**. For dark and photo-fermentation system, an increase in initial investment, reagents consumption, welfare, and water/power charge would cause a decrease in the NPV. The rising hydrogen and fermentation effluent selling prices would also raise the NPV. When hydrogen price goes from -30% to +30%, the financial NPV changes from 50,000 CNY to 1,118,800 CNY for dark and photo- fermentation system.

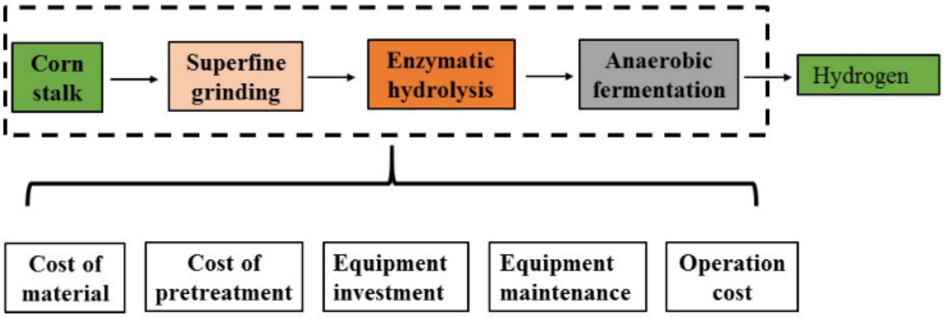

Supplementary Fig.13. Transformation process of straw biomass based hydrogen production system

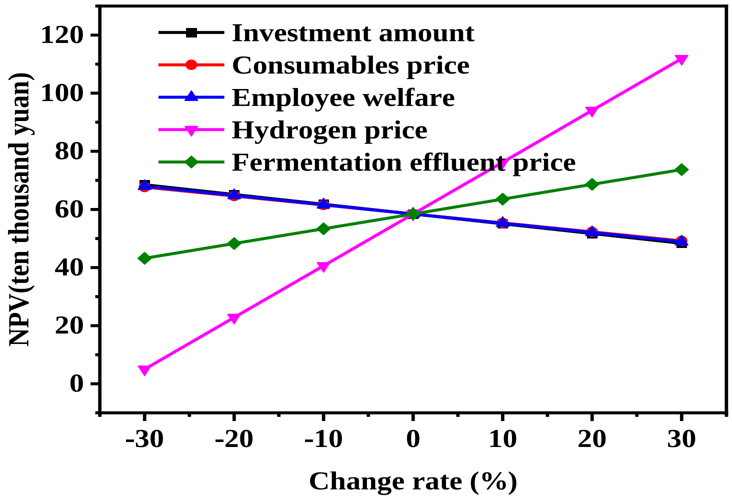

Supplementary Fig.14. Relation between NPV and the related factors

Supplementary Table.3. Cost of hydrogen from different hydrogen production technology

Hydrogen production technology	Cost ($/kg)	Reference
Water electrolysis (onshore wind power,)	7.25	[14]
Water electrolysis (Onshore photovoltaics)	11.77	[14]
Water electrolysis (wind power)	8.87	[15]
Water electrolysis (photovoltaics power)	6.33	[15]
Biomass gasification	4.11	[16]
Glycerol reforming	7.45	[17]
Dark- and photo-fermentation	5.6	In the study

Supplementary Table.4. Profit and loss statement and dynamic economic analysis

Project	Dark-photo fermentation
Sales revenue (CNY)	272397.6
Total cost (CNY)	157950.56
Income tax (CNY)	11444.70
Net profit (CNY)	103002.34
NPV (10^4 CNY)	58.44
Tp (Year)	6.86
IRR(%)	16.84

Reference

1. Li, Y. *et al.* Comparison of bio-hydrogen production yield capacity between asynchronous and simultaneous saccharification and fermentation processes from agricultural residue by mixed anaerobic cultures. *Bioresour. Technol.* **247**, 1210–1214 (2018).

2. Zhang, Z. *et al.* Investigation of the interaction between lighting and mixing applied during the photo-fermentation biohydrogen production process from agricultural waste. *Bioresour. Technol.* **312**, 123570 (2020).

3. Wang, Z. *et al.* Life cycle environmental impacts of cornstalk briquette fuel in China. *Appl. Energy* **192**, 83–94 (2017).

4. Wang, Z., Li, L. & Zhang, G. Life cycle greenhouse gas assessment of hydrogen production via chemical looping combustion thermally coupled steam reforming. *J. Clean. Prod.* **179**, 335–346 (2018).

5. Verma, A. & Kumar, A. Life cycle assessment of hydrogen production from underground coal gasification. *Appl. Energy* **147**, 556–568 (2015).

6. Liu, H., Guo, W., Fan, Z., Huang, F. & Liu, S. Comparative life cycle energy, water consumption and carbon emissions analysis of deep in-situ gasification based coal-to-hydrogen with carbon capture and alternative routes. *J. Clean. Prod.* **426**, 139129 (2023).

7. Susmozas, A., Iribarren, D., Zapp, P., Linβen, J. & Dufour, J. Life-cycle performance of hydrogen production via indirect biomass gasification with CO 2 capture. *Int. J. Hydrogen Energy* **41**, 19484–19491 (2016).

8. Cetinkaya, E., Dincer, I. & Naterer, G. F. Life cycle assessment of various hydrogen production methods. *Int. J. Hydrogen Energy* **37**, 2071–2080 (2012).

9. Hajjaji, N., Pons, M. N., Renaudin, V. & Houas, A. Comparative life cycle assessment of eight alternatives for hydrogen production from renewable and fossil feedstock. *J. Clean. Prod.* **44**, 177–189 (2013).

10. Zhang, X., Bauer, C., Mutel, C. L. & Volkart, K. Life Cycle Assessment of

Power-to-Gas: Approaches, system variations and their environmental implications. *Appl. Energy* **190**, 326–338 (2017).

11. Gerloff, N. Comparative Life-Cycle-Assessment analysis of three major water electrolysis technologies while applying various energy scenarios for a greener hydrogen production. *J. Energy Storage* **43**, 102759 (2021).

12. Meky, N., Ibrahim, M. G., Fujii, M. & Elreedy, A. Integrated dark-photo fermentative hydrogen production from synthetic gelatinaceous wastewater via cost-effective hybrid reactor at ambient temperature. *Energy Convers. Manag.* **203**, 112250 (2020).

13. Hay, J. X. W., Wu, T. Y., Juan, J. C. & Md. Jahim, J. Improved biohydrogen production and treatment of pulp and paper mill effluent through ultrasonication pretreatment of wastewater. *Energy Convers. Manag.* **106**, 576–583 (2015).

14. Shin, H. *et al.* Techno-economic evaluation of green hydrogen production with low-temperature water electrolysis technologies directly coupled with renewable power sources. *Energy Convers. Manag.* **286**, 117083 (2023).

15. Hassan, Q., Sameen, A. Z., Salman, H. M. & Jaszczur, M. Large-scale green hydrogen production via alkaline water electrolysis using solar and wind energy. *Int. J. Hydrogen Energy* **48**, 34299–34315 (2023).

16. Cook, B. & Hagen, C. Techno-economic analysis of biomass gasification for hydrogen production in three US-based case studies. *Int. J. Hydrogen Energy* (2023) doi:10.1016/j.ijhydene.2023.07.219.

17. Khodabandehloo, M., Larimi, A. & Khorasheh, F. Comparative process modeling and techno-economic evaluation of renewable hydrogen production by glycerol reforming in aqueous and gaseous phases. *Energy Convers. Manag.* **225**, 113483 (2020).
